# Formation and Physiochemical Properties of Silver Nanoparticles with Various Exopolysaccharides of a Medicinal Fungus in Aqueous Solution

**DOI:** 10.3390/molecules22010050

**Published:** 2016-12-29

**Authors:** Wenjie Jian, Lu Zhang, Ka-Chai Siu, Angxin Song, Jian-Yong Wu

**Affiliations:** 1 Department of Applied Biology & Chemical Technology, State Key Laboratory of Chinese Medicine and Molecular Pharmacology in Shenzhen, The Hong Kong Polytechnic University, Hung Hom, Kowloon, Hong Kong, China; jianwenjie123@163.com (W.J.); lzhang17@gmail.com (L.Z.); kcsiu@polyu.edu.hk (K.-C.S.); ang-xin.song@connect.polyu.hk (A.S.); 2 Department of Medical Technology, Xiamen Medical College, Xiamen 361000, China

**Keywords:** silver nanoparticle, fungal exopolysaccharide, molecular characteristics, pH, dispersion stability, molecular interactions

## Abstract

Natural polysaccharides are the most widely used biopolymers for green synthesis of eco-friendly silver nanoparticles (AgNPs). In a previous study, a high molecular weight (MW) fraction of exopolysaccharides (EPS) produced by a medicinal fungus Cs-HK1 has been shown useful for green and facile synthesis of AgNPs in water. This study was to further evaluate the effects of molecular properties of EPS on the formation, stability and properties of AgNPs with different EPS fractions at various pH conditions. Three EPS fractions (P_0.5_, P_2.0_ and P_5.0_: MW high to low and protein content low to high) were reacted with silver nitrate at various pH 3.0–8.0 in water. The most favorable pH range was 5.5–8.0 for the formation and stable dispersion of AgNPs. At a given pH, the maximum amount of AgNPs was produced with P_5.0_, and the minimum with P_0.5_. The shape, size and physiochemical properties of AgNPs were strongly affected by the molecular characteristics of EPS (MW and conformation). The results may be helpful for understanding the factors and mechanisms for formation of stable AgNPs with natural polysaccharides and the interactions between AgNPs and the polysaccharide hydrocolloids in water.

## 1. Introduction

Silver nanoparticles (NPs) have a wide range of potential applications because of their unique properties and bioactivities [1]. A common and conventional approach for preparation of AgNPs is via the reaction of silver nitrite (AgNO_3_) with a chemical reducing agent such as sodium borohydride and sodium citrate, followed by the addition of a polymer as a stabilizing or capping agent. Recently, natural polysaccharides from various sources have been widely applied to replace the chemical agents for green or eco-friendly syntheses of AgNPs [2,3]. As some of these polysaccharides may act as both reducing and stabilizing agent, the synthesis can be accomplished in a single step [4,5]. Many previous studies have evaluated the use of microbial exopolysaccharides (EPS) for preparation of AgNPs, such as xanthan [6], dextran [7], gellan [8], curdlan [9], and alginate [10]. Although these methods are quite simple and eco-friendly, a lot more work is needed to investigate the conditions and reaction mechanisms for the synthesis of AgNPs with the desired morphology and composition.

Despite of the potential advantages offered by the synthesis of AgNPs with the microbial EPS, there is still a need to develop reliable and practical methods for the rational control of the reaction conditions to attain desired nanoparticles with the complex structures and hydrocolloidal behaviors of EPS in water [11]. For example, Bankura et al. prepared dextran-based AgNPs with a wide distribution in diameter [12], and Liu et al. prepared highly stable AgNP dispersions in water using sodium alginate under gamma radiation [10]. However, most previous studies have been concerned with the reaction conditions, particle morphology and anti-bacterial activity [13,14]. Few have addressed the relationships between the molecular characteristics and hydrocolloidal behaviors of EPS and the formation and stability of AgNPs in aqueous solutions [15,16]. The specific relationship between the chemical structure of EPS and the physicochemical properties of AgNPs is not clear [17].

In a previous study from our group, AgNPs were successfully made with a high molecular weight (MW) polysaccharide-protein complex fraction isolated from exopolysaccharide (EPS) liquid fermentation Cs-HK1 fungus [18]. The AgNPs retained a stable dispersion in EPS solution and exhibited significant growth inhibition of both Gram-negative and -positive bacteria but a very low cytotoxicity to the RAW264.7 macrophage cells.

Because the EPS acted as both reducing and capping agent in AgNP synthesis, its chemical and physical properties may affect the formation and properties of AgNPs. Solution pH is an important factor affecting the aggregation and dispersion of AgNPs in EPS because of its influence on surface charge of EPS molecules. This study was to gain better understanding of the effects of polysaccharide molecular properties on the synthesis and stability of AgNPs in an aqueous solution. AgNPs were synthesized using three different EPS fractions from the Cs-HK1 fungus under various pH conditions and the physiochemical properties of the AgNP dispersion were measured.

## 2. Results

### 2.1. Chemical Composition of EPS Fractions

Table 1 summarizes the chemical composition and molecular properties of three EPS fractions. All three EPS fractions consisted of both polysaccharide and protein but in different ratios. The polysaccharide to protein ratio (*w*/*w*) was 93:7, 69:31, and 54:46 for P_0.5_, P_2.0_ and P_5.0_, respectively. The EPS fraction P_0.5_ was composed mainly of polysaccharide with a small fraction of protein, while the P_2.0_ and P_5.0_ were either mixtures or complexes of polysaccharide and protein. The composition change of EPS fractions attained with gradient ethanol precipitation of Cs-HK1 mycelial fermentation medium was consistent with that in our previous study [19]. 

The monosaccharide analysis of the EPS fractions further revealed that the polysaccharides in all three EPS fractions were composed of mannose, glucose, and galactose (Figure S1). The molar ratio of mannose, glucose, and galactose was 1.3:10.8:1, 16:1:7.3 and 15:1:15.5 for the polysaccharides in P_0.5_, P_2.0_, and P_5.0_, respectively. Glucose was the major component of P_0.5_. In contrast, P_2.0_ and P_5.0_ had significantly higher mannose and galactose contents than P_0.5_. Following similar procedure used in this study, Chen et al. [20] obtained a galactomannan-protein complex from purification of a lower molecular EPS fraction precipitated with a relatively high ethanol volume from the fermentation broth of Cs-HK1. The chemical composition of P_2.0_ and P_5.0_ indicated that galactomannan-protein complex is most likely to be the major component of these two EPS fractions.

### 2.2. Molecular Characteristics of EPS Fractions

Large differences were also detected in the molecular characteristics of the three EPS fractions as determined by GPC-MALLS-UV. EPS fractions showed a steady decrease in their weighted-average molecular weight (MW), with the gradual increase in the ethanol volume used in the precipitation. The sugar rich fraction P_0.5_ had the highest MW of 1022 kDa. The MW of P_2.0_ and P_5.0_, 85.16 kDa and 21.67 kDa, were significantly smaller. As expected, the root mean square radius (Rm) of the three EPS fractions correlated with their MW (Table 1). In contrast, the three EPS fractions demonstrated a different trend in the polydispersity (PD), which describes the width of the molecular weight distribution. The relatively low MW fraction P_2.0_ (with a PD of 10.8) was the most heterogeneous EPS fraction, whereas the PD of the other two fractions P_0.5_ and P_5.0_ was 5.170 and 3.287. Given that the PD values of the three EPS fractions far exceeded unity, all three EPS fractions contain molecules with a range of different MWs. 

The GPC-MALLS analysis enables the estimation of the molecular conformation of EPS fractions from the slope of double logarithmic plot of Rm versus molecular weight [21]. The slope for P_0.5_ matches the expected value for a random coil (0.33). Both P_2.0_ and P_5.0_ had a slope less than the value for a sphere (0.33), indicating they exist in sphere-like shape and maintain a much more compact structure than P_0.5_ in aqueous solution. 

Moreover, MALLS analysis characterizes the second virial coefficient (A_2_), which describes the interactions of macromolecules. The value of A_2_ quantifies the degree of intermolecular interactions of macromolecules in dilute solutions due to molecular forces including ionic, hard-sphere, van der Waals and other short-range interactions (e.g., hydrophobic interactions) [22]. A positive A_2_ value indicates repulsive interactions between molecules, whereas a negative A_2_ value represents attractive intermolecular interactions. The A_2_ value is negative for P_0.5_ and positive for P_2.0_ and P_5.0_. These results suggest that the EPS fraction P_0.5_ tends to form aggregates or precipitate after heating or long storage due to the attractive intermolecular interactions. In addition, the repulsive molecular interactions in P_2.0_ and P_5.0_ allow them to retain a stable dispersion in solution. The larger A_2_ of P_5.0_ than P_2.0_ further indicated stronger repulsive interactions in P_5.0_ than P_2.0_.

In general, the high MW P_0.5_ was significantly different from the low MW fractions P_2.0_ and P_5.0_ in terms of their chemical composition and molecular characteristics. The molecular conformation of the EPS fractions may affect the accessibility of the functional groups that reduces silver ions to AgNPs and interacts with AgNPs to stabilize them in an aqueous solution. The molecular interactions may also affect the ability of the EPS fraction to attract silver ions and the stability of the suspension of complexes formed between EPS fraction and AgNPs. As a result, a drastic variation is expected in the performance of P_0.5_, and P_2.0_ and P_5.0_ as the reducing and capping agent for AgNP synthesis.

### 2.3. Optical Properties of AgNP Dispersions

The interaction between AgNPs and light makes it possible to monitor the formation of AgNPs. The reduction of silver ion to silver atom is accompanied by a change in the color of AgNO_3_ solution to yellow, as a result of the surface plasmon resonance (SPR) of AgNPs. The SPR can be detected as an absorption band using UV-Vis spectroscopy [23]. The intensity of the absorption band correlates with the AgNP concentration [24], whereas the wavelength and width of the SPR band are related to the size and shape of the AgNPs [25]. Therefore, the UV-Vis spectral properties of AgNPs provide a mechanism for detecting and characterizing AgNPs in aqueous solution.

**Figure 1:**
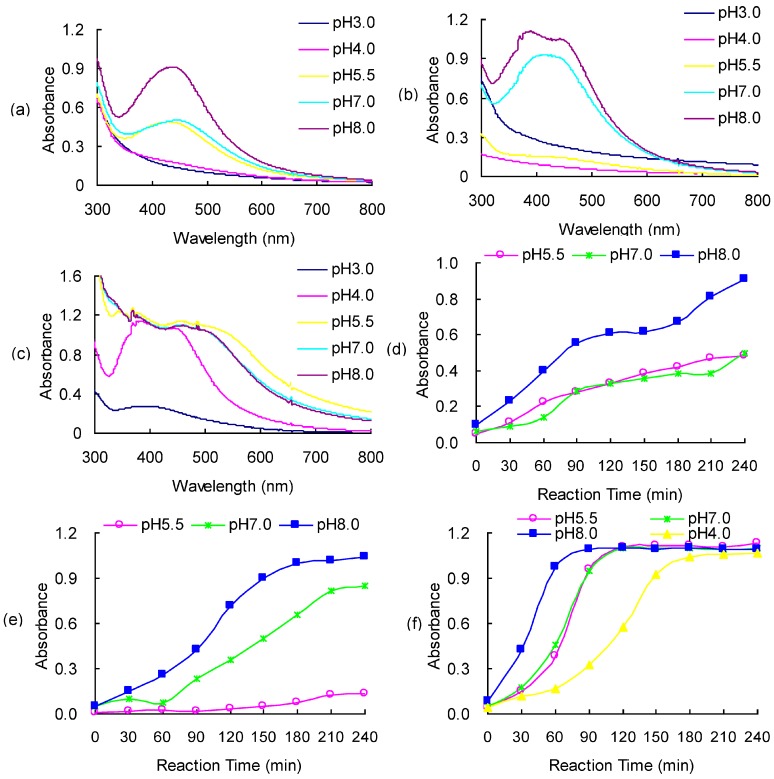
UV-Vis absorbance spectra of AgNO_3_ and (**a**) P_0.5_; (**b**) P_2.0_; (**c**) P_5.0_ mixture reaction solution at various pH value after thermal treatment for 240 min; and peak absorbance at UV-Vis light absorbance spectra of AgNO_3_; and (**d**) P_0.5_; (**e**) P_2.0_; (**f**) P_5.0_ mixture reaction solution at various pH value for various periods of reaction time.

Figure 1 shows the UV-Vis spectra of AgNO_3_ and EPS fractions (a) P_0.5_, (b) P_2.0_ and (c) P_5.0_ mixture reaction solutions at various pH values prepared at 100 °C for 240 min. It was observed that high pH induced precipitation of black particles in the reaction mixture of AgNO_3_ and EPS (Figure S2), which is due to formation of Ag_2_O [26]. Therefore, reaction mixtures at pH 9.0 and 10.0 were excluded from UV-Vis absorbance measurement for all EPS fractions. For the P_0.5_ and AgNO_3_ mixture reaction solutions (P_0.5_-AgNO_3_), the pH of the reaction mixture had a significant effect on the formation of AgNPs (Figure 1a). At low pH values (3.0 and 4.0), no absorption band was observed in the measured wavelength range. When the pH reached 5.5, one small peak at approximately 440 nm was detected (Figure 1a). With the increase in the pH from 5.5 to 8.0, the intensity of the absorption peak increased. These results suggested that reduction of Ag ion to AgNPs by EPS fraction P_2.0_ only occurred when the pH is at or above 5.5. A higher pH favored the formation of AgNPs, leading to a higher concentration of AgNPs in the solution.

The dependence of AgNP formation on the pH condition was also observed in the reaction mixture of AgNO_3_ and the other two EPS fractions. For the mixture of P_2.0_ and AgNO_3_ (P_2.0_-AgNO_3_), a weak absorption band was detected at pH 5.5 (Figure 1b). The intensity and width of the band increased with further increase in the pH to 7.0 and 8.0. At pH 8.0, the absorption band of the mixture changed from a single broad peak to a narrow peak at round 390 nm with a hump near 460 nm. The SPR absorption band of AgNPs is sensitive to physical state of the particles, such as the size and shape. It has been reported that an additional quadrupole resonance peak can be caused by an increase in the particle size of AgNPs or aggregation of AgNPs [25]. As indicated by the presence of the quadrupole resonance peak near 390 nm at an alkaline condition (pH 8), the further increase in pH of the reaction mixture can facilitate the particle growth or aggregation of AgNPs. 

For the mixture of P_5.0_ and AgNO_3_ (P_5.0_-AgNO_3_), a weak absorption band occurred near 400 nm even at an acidic condition (pH 3.0) (Figure 1c). At pH 4.0, a quadrupole resonance peak was detected near 370 nm in addition to the dipolar resonance peak at 450 nm. With the increase in pH, the peak at 450 nm red shifted and the intensity of the two peaks increased slightly. This red shift suggested an increase in the size of AgNPs or a decrease in the space among AgNPs with the increase in pH. Besides the physical properties and yield of the AgNPs, the pH of the reaction mixture also affected the rate of the reduction reaction. The intensity of the SPR absorption peak was recorded periodically during the thermal treatment of EPS-AgNO_3_ solutions (Figure S3). The increase in pH shortened the time needed for noticeable increment in the SPR absorption peak intensity, implying an increase in the reaction rate of silver ions.

The UV/Vis absorbance results were consistent with the color change in the reaction mixture of AgNO_3_ and EPS fractions (Figure S3). The onset of color change from transparent to yellow occurred at pH 5.5, pH 7.0, and pH 4.0 for the EPS-AgNO_3_ solution containing P_0.5_, P_2.0_, and P_5.0_, respectively, which suggested the onset of formation of AgNPs. For all EPS fractions, the yellow color was intensified as the pH increased. On the other hand, the color was also affected by the EPS fraction in the reaction mixture. The yellow color of the solution intensified with the decrease in the MW of the EPS fraction, in the order of P_0.5_, P_2.0_, and P_5.0_. The P_5.0_-AgNO_3_ mixture displayed a dark brown color at pH 8.0. Similar to the SPR absorption band, the color of AgNPs solution is affected by its size, shape, and concentration. The intensified color was probably attributed to a higher AgNP concentration in the solution. 

Previous studies have shown the promoting effect of alkaline condition on AgNP synthesis with various reducing agents [25,27]. The alkaline condition facilitates the deprotonation of functional groups on EPS fractions. The more negatively charged EPS fractions are capable of attracting more Ag^+^ and hence increase the chance of the reduction reaction of Ag^+^. Moreover, hydroxyl ions are involved in the reduction of silver ions [27]. The alkaline solution provided excessive hydroxyl ions promoting the reduction of silver ions. On the other hand, pH had different effects on the reduction of Ag^+^ by the three EPS fractions. As shown in Figure 1, formation of AgNP occurred in the P_5.0_-AgNO_3_ mixture even at an acidic condition. Under alkaline condition, P_5.0_-AgNO_3_ demonstrated the highest yield of AgNPs as well. The low MW EPS fraction P_5.0_ may possess more silver ion binding functional groups on its surface than higher MW P_0.5_ and P_2.0_. In addition, protein molecules contain amino acid moieties that favor metal reduction, such as tyrosine, arginine, cysteine, methionine, and lysine [28]. The polysaccharide-protein complex P_5.0_ gained more reducing power from its higher protein content. Huang et al. [19] measured the ferric reducing ability of different EPS fractions extraction from Cs-HK1 fermentation medium. They found that the polysaccharide-protein complex with low MW demonstrated highest reducing power and the reducing power dropped with the decrease in the protein content of the EPS fraction.

### 2.4. Characteristics of EPS-AgNP Complexes

Following the reduction of silver ions to elemental silver and the formation of AgNPs, the AgNPs interact with the EPS molecules to form AgNP-EPS complexes, preventing their aggregation and precipitation in the solution. The combination of EDS and TEM made it possible to measure the composition of the AgNP-EPS complexes directly in the solution. The TEM-EDS analysis was performed only on reagent solutions with detected AgNP formation, i.e., P_0.5_-AgNO_3_ (pH 5.5, 7.0, and 8.0), P_2.0_-AgNO_3_ (pH 5.5, 7.0, and 8.0)_,_ and P_5.0_-AgNO_3_ (pH 4.0, 5.5, 7.0, and 8.0).

Based on the chemical composition of EPS fractions, the EPS-AgNP complexes should consist of four elements, namely, carbon, nitrogen, oxygen, and silver. As expected, the EDS analysis revealed that P_0.5_-AgNP, P_2.0_-AgNP, and P_5.0_-AgNP were composed of these four elements in different ratios (Table 2). The EDS spectrum showed a fifth peak corresponding to copper (Figure S4), which was background noise from the copper mesh grid used for EDS measurement. Silver was the primary component in all EPS-AgNP complexes. Depending on the pH and EPS composition, the silver content ranged from 56.4% (*w*/*w*) to 81.5% (*w*/*w*) of the total EPS-AgNP weight. Similar to the observation with UV spectrometer (Section 2.3), the silver content increased with the increase in pH for each EPS-AgNP complex (Table 2). The EPS used for AgNP synthesis had a much significant effect on the silver content of EPS-AgNP. The highest silver content (78.5%–81.5%) was obtained in the complex prepared with the low MW protein rich P_5.0_. The high MW sugar rich P_0.5_ led to the production of EPS-AgNP with the lowest silver content (56.4%–58.2%). These observations demonstrated that EPS with more protein component and lower MW acted as a better capping agent in addition to its better reducing power.

In our previous study, Fourier transform infrared (FTIR) spectrometry analysis was performed of the AgNPs formed in the EPS solution [18], suggesting the binding of AgNPs to the hydroxyl groups of EPS. The smaller P_2.0_ and P_5.0_ fractions may contain more hydroxyl groups per molecule on the surface, forming a stronger interaction between AgNPs and the EPS molecules.

**Figure 2:**
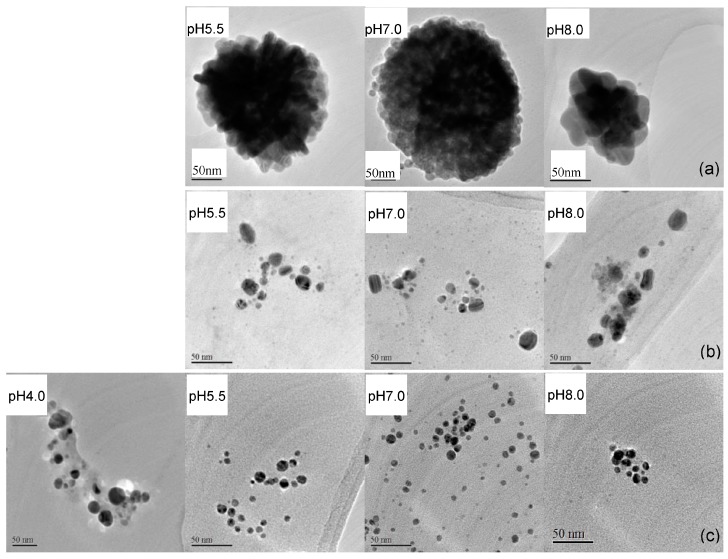
Transmission electron microscopy of (**a**) P_0.5_-AgNPs; (**b**) P_2.0_-AgNPs; and (**c**) P_5.0_-AgNPs at different pH. (AgNP formation did not occur at pH = 4 for EPS fractions P_0.5_ and P_2.0_).

The TEM images of EPS-AgNP complexes are shown in Figure 2. Because the contrast of EPS fractions was much lower than AgNPs, the captured morphological properties in TEM images were attributed to those of AgNPs in EPS-AgNP complexes. Significant differences were observed in the morphology and size of AgNPs prepared with different EPS fractions under various pH conditions. The type of EPS fraction used in AgNP synthesis had a more distinct effect on morphological characteristics of AgNPs comparing to the effect of pH. At all pH conditions, the P_0.5_-AgNPs presented as globular clusters composed of relatively uniformly distributed spherical silver nanoparticles (Figure 2a). The boundary of each single particle was clearly visible in the TEM images. The pH of the solution had small but noticeable effect on the particle size of individual AgNPs. At pH 8, the average diameter of the AgNPs increased to 22 nm from approximately 17 nm at pH 5.5 and 7.0. Interestingly, the globular AgNP cluster did not cause an additional quadrupole resonance in the UV-Vis spectrum due to the decreased space between AgNPs. The random coil confirmation of P_0.5_ enables it to attain negatively charged functional groups, e.g., hydroxyl groups, both on the surface of the molecules and in the gaps created by the random chain folding. The formation of the AgNP clusters was caused by attachment of individual AgNPs to these sites. As a result, physical barriers are very likely to exist in between AgNPs even though the AgNPs appeared as aggregates visually. 

The large globular clusters were not present in the P_2.0_-AgNP and P_5.0_-AgNP samples. In the P_2.0_-AgNPs solutions (Figure 2b), AgNPs dispersed in the solution as spherical particles with an average size of 10 nm at pH 5.5. The AgNPs size increased slightly to 12 nm at pH 7.0. Further increase in the pH to 8.0 caused spherical AgNPs to aggregate into irregular shaped, e.g., rectangular-like and oval-like, particles with the long dimension of 20 nm. The formation of small aggregations explains the emerging of the quadrupole resonance peak in UV-Vis spectrum of the P_2.0_-AgNPs solution at pH 8.0. In P_5.0_-AgNPs, AgNPs existed mainly as spherical particles smaller than P_0.5_-AgNPs and P_2.0_-AgNPs on average (Figure 2c). The particle size of P_5.0_-AgNPs was affected by the pH of the solution as well. At pH 4.0, the AgNPs had a wide particle size distribution ranging from 15 nm to 30 nm. Some irregular shaped particles were produced as a result of the slow nucleation and growth process caused by slow reduction rate at low pH [29]. At higher pH values, 5.5, 7.0, and 8.0, the AgNPs adopted a more regular spherical shape. The average particle size dropped significantly to around 10 nm at pH 5.5, and remained relatively constant with further increase in pH (Figure 2c). These results have further demonstrated the effect of the molecular properties of EPS and solution pH on the composition and morphology of AgNPs and EPS-AgNPs. The particle size of AgNPs in P_5.0_-AgNPs was much smaller than that in previous studies [4,12], due probably to the differences in molecular properties of polysaccharides. Other than the spherical AgNPs obtained in the present study, triangle, square and various other shapes of AgNPs have been reported in the literature [30,31]. Generally, in growth-directed preparation, the shape of metallic NPs is dependent on several factors such as metal precursor, reducing agent, protecting agent and controlling reaction kinetics [32]. 

TEM with selected area electron diffraction pattern (SAED) on the AgNPs, is an effective alternative to other techniques such as X-ray diffraction (XRD) pattern measurement for detecting the valence state of metallic element [33]. Several previous studies have used SAED to determine the presence of AgNPs [34,35,36]. As displayed in the SAED of EPS-AgNP complexes (supplemental data), only a few spots of diffraction appeared on concentric circles, and the ring patterns are consistent with the plane families of pure face-centered silver structure. These observations convinced that only silver nanoparticles and no silver oxide nanoparticles were present in the samples and is also consistent with the literature report [37]. Similarly, silver oxide nanoparticles were not found in the AgNPs prepared with microbial EPS (using XRD) in previous studies [23,38]. In contract, mainly silver oxide nanoparticles were found by XRD during the biosorption of silver ions by bacterial EPS at room temperature [39,40]. Such differences may be due to the different experimental conditions especially the temperature since thermal treatment favors the reduction of Ag^+^ to Ag° [41].

### 2.5. Hydrodynamic Radii of EPS-AgNP Complexes

**Figure 3:**
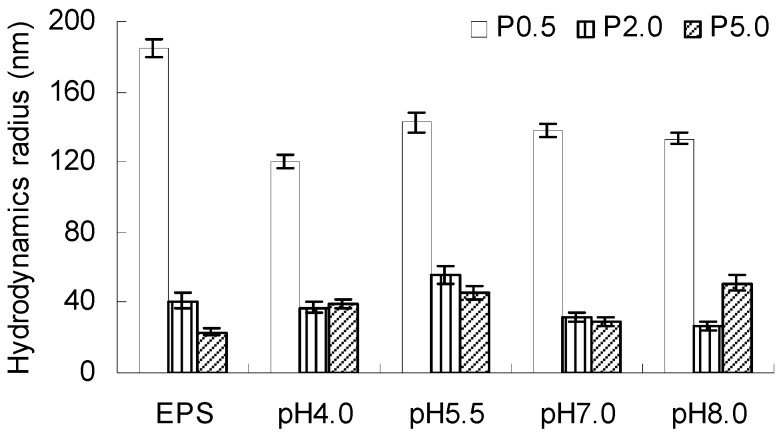
Hydrodynamic radius of EPS fractions (P_0.5_, P_2.0_, P_5.0_) and EPS-AgNP complexes (P_0.5_-AgNPs, P_2.0_-AgNPs, P_5.0_-AgNPs) at different pH. (AgNP formation did not occur at pH = 4 for EPS fractions P_0.5_ and P_2.0_).

Figure 3 shows the hydrodynamic radius of EPS fractions and EPS-AgNP complexes at different pH values. The size of EPS-AgNP complexes was dependent on the size of the EPS fraction in the complexes. P_0.5_ had an Rh larger than P_2.0_ and P_5.0_. Similarly, P_0.5_-AgNP complex had the largest Rh among the three different types of EPS-AgNP. The DLS measurement of Rh tells the size of the EPS-AgNP complexes instead of the AgNPs alone.

The Rh of P_0.5_-AgNP was in the range of 120.4 nm–142.78 nm at different pH values, which is a drastic drop from the original size of P_0.5_ (142.78 nm). The decrease in the size of EPS was caused by the shrinkage of polysaccharide molecules after capping nano-metal particles [42]. The random coil conformation of EPS fraction P_0.5_ determined that the molecules are loosely packaged chains. The AgNPs attached to the negatively charged functional groups on the P_0.5_ molecule modified the electrostatic interaction within the molecule, thereby altering the chain packing in P_0.5_ molecules. Similar trend was found in P_2.0_ except for an increase at pH 5.5. The P_2.0_-AgNP complex was smaller in Rh at pH 7.0 and 8.0 than P_2.0_ itself. The sphere like P_2.0_ molecules had a more compact structure than P_0.5_. Because pH can affect the surface charge of macromolecules significantly, the structure of P_2.0_ may become less compact as a result of the change in the electrostatic interaction within the molecule at relatively higher pH. The less compact spherical P_2.0_ may experience a change in its secondary structure due to the interaction with AgNPs, leading to the smaller molecular size. On the other hand, P_5.0_ demonstrated a different behavior from P_0.5_ and P_2.0_. After capping AgNPs, the generated P_5.0_-AgNP complexes had Rh values in the range of 28.9–51.0 nm at different pH conditions. At pH 8.0, the Rh of P_5.0_-AgNP complexes almost doubled the Rh of P_5.0_. The variable effect of the capping process on the size of EPS fractions suggests that different mechanisms were involved in the capping of nanoparticles by different EPS fractions.

### 2.6. Stability of EPS-AgNP Dispersions

**Figure 4:**
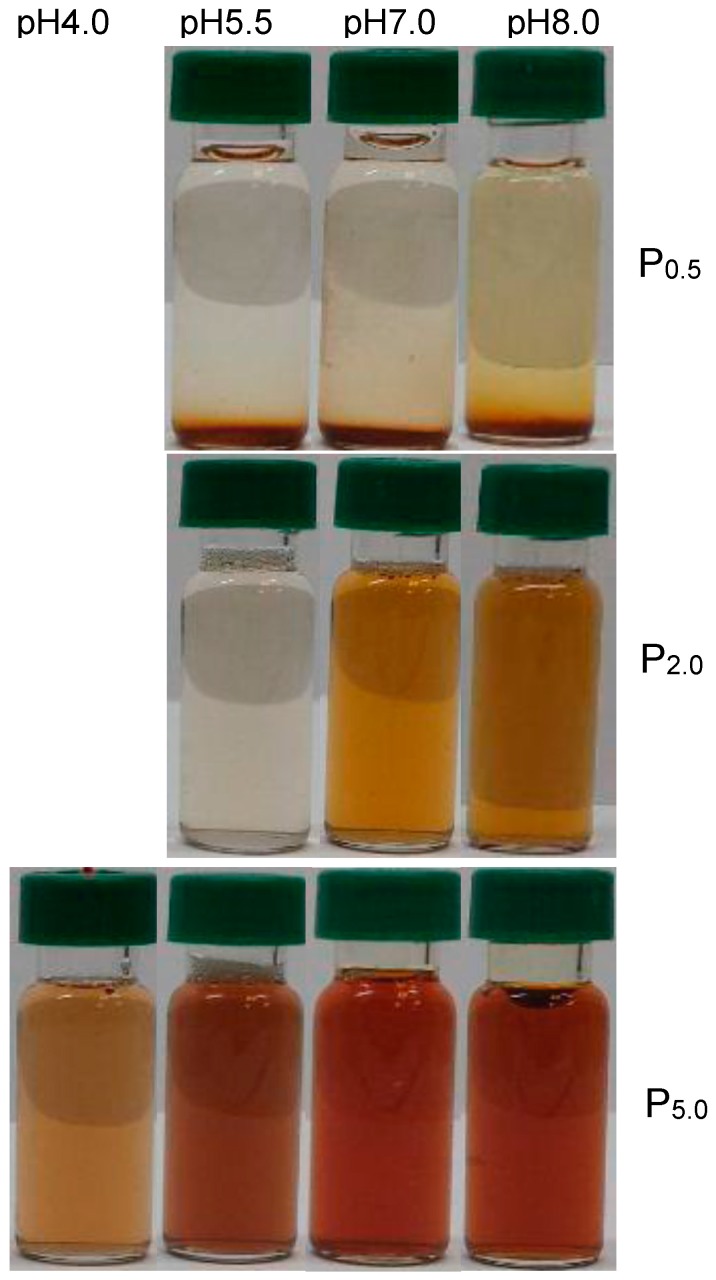
Photographs of EPS-AgNP dispersions at pH 4.0, pH 5.5, pH 7.0, and pH 8.0 after stored at room temperature for 2 months. (AgNP formation did not occur at pH = 4 for EPS fractions P_0.5_ and P_2.0_).

The stability of AgNPs dispersion is an important property affecting their biological and medical applications. Because the Rh directly affects the colloidal behavior and dispersion stability of nanoparticles, the large Rh of P_0.5_-AgNPs may disrupt the stability of its suspension. The dispersion stability of the EPS-AgNP complexes was further investigated by monitoring the samples for two months. The photos for EPS-AgNP dispersions stored at room temperature for 2 months were shown in Figure 4. Extensive precipitate of brown particles was observed in P_0.5_-AgNP solutions at all pH values conditions, whereas P_2.0_-AgNP and P_5.0_-AgNP sample solutions retained their optical properties and stayed as stable dispersions irrespective of the pH. As expected, the large P_0.5_-AgNP complexes tended to form aggregates, which destabilized the suspension of the complexes and lead to poor long term dispersion stability. Besides the intrinsic properties of nanoparticles, the capping agents can exert a strong influence on the characteristics of the capped nanoparticles [23,24]. The aforementioned second virial coefficients of EPS fractions (Section 2.2) proved that P_0.5_ molecules possessed intermolecular attractive forces driving the formation of aggregation during long term storage. The repulsive interactions in P_2.0_ and P_5.0_ allowed them to maintain their stability in aqueous solution during storage. It is evident that the dispersion stability of the capping agent EPS is a major contributor to the stability of EPS-AgNP dispersion.

### 2.7. Mechanisms of EPS—AgNP Complex Formation

**Figure 5:**
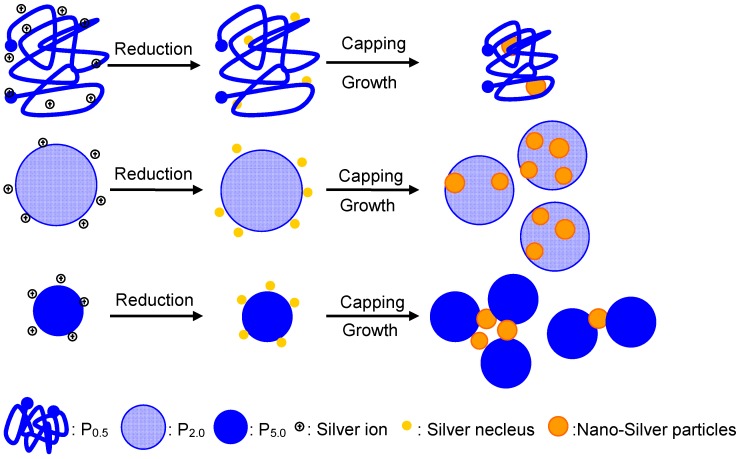
Schematic model for formation of EPS-AgNP complexes.

Based on the above characteristics of EPS fractions and EPS-AgNP complexes, we propose a mechanistic model for the formation of AgNPs and their interaction with EPS in EPS solution (Figure 5). When the precursor AgNO_3_ was added to the EPS aqueous solution, the silver ions (Ag^+^) were reduced to silver atoms by the reducing groups on EPS such as sulfydryl, amino, hydroxyl and the attached small molecules at elevated temperature. After that, the produced silver atoms served as nucleii and grew gradually into AgNPs upon further supply of silver atoms from the reduction reaction. In the case of the studies EPS fractions, the protein rich EPS P_5.0_ offered more sites to attract and reduce silver ions than P_0.5_ and P_2.0_. Because these sites provided potential locations for nucleation of AgNPs, more AgNPs were formed in the P_5.0_ solution than P_0.5_ and P_2.0_ as supported by the UV-Vis results and the chemical composition of the EPS-AgNP complexes. In the growth process, small neighboring AgNPs coalesce into larger particles [25]. On the other hand, the EPS molecules can capture and interaction with the AgNPs and thus control the growth and aggregation of AgNPs through steric or electrosteric repulsions [26]. Therefore, the small AgNPs involved in the coalescence may be attached to adjacent chains in the same EPS molecule or multiple EPS molecules. As a result, the coalescence of AgNPs leads to intramolecular association or intermolecular agglomeration of EPS molecules. During the formation of P_0.5_-AgNP and P_2.0_-AgNP, the growth of AgNPs may occur mainly within each EPS molecule. Such intramolecular association may explain the shrinkage of the EPS molecules in the P_0.5_-AgNP and P_2.0_-AgNP complexes. In P_5.0_-AgNPs, the low MW P_5.0_ molecules were brought together by the clustering of AgNPs, forming larger P_5.0_-AgNP complexes.

## 3. Materials and Methods

### 3.1. Fungal Fermentation

The Cs-HK1 fungus was isolated from a natural *Cordyceps sinensis* fruiting body and the Cs-HK1 stock culture was maintained on potato dextrose agar (PDA) solid medium at 20 °C in Petri dishes. Shake-flask culture was initiated by inoculating the mycelial spores from the stock culture into 250 mL Erlenmeyer flasks each containing 50 mL liquid culture medium which was composed of 40 g/L glucose, 10 g/L yeast extract, 5 g/L peptone, 1 g/L KH_2_PO_4_ and 0.5 g/L MgSO_4_·7H_2_O. The flasks were maintained in a shaking-incubator at 150 rpm and 20 °C for 7 days and the mycelial broth (400 mL) was then added into a 15 L stirred aerated fermenter (Biostat^®^C, Satorius, Göttingen, Germany) filled with 8 L of liquid culture medium. The fermentation was operated for 6 days at 20 °C, 1 vvm air flow rate, and dissolved oxygen above 20% air saturation. At the end, the mycelial broth was centrifuged to obtain a solid free supernatant for the following EPS isolation. 

### 3.2. Preparation of EPS Fractions

Gradient ethanol precipitation method was used to isolate EPS factions in different MW ranges from the mycelial culture medium as reported previously [19]. Ethanol (95% grade) was slowly added to the liquid medium to achieve an ethanol-to-liquid medium volume ratio of 0.5. The mixture was stirred for 3 h at room temperature and kept stationary at 4 °C for overnight. After the mixture was centrifuged at 12,000 rpm for 20 min, the precipitate was collected as the EPS fraction P_0.5_. The supernatant was subjected to another step of precipitation with a higher ethanol-liquid medium volume ratio using the procedure described above for P_0.5_. With the same method, two more precipitated fractions, P_2_, and P_5_, were collected successively at 2 and 5 ethanol-to-liquid medium volume ratios in addition to P_0.5_. All three EPS precipitates were freeze-dried and stored at 20 °C–25 °C for further analysis and experiments.

### 3.3. Composition Analysis and Characterization of EPS Fractions

The sugar and protein content of the EPS fractions were determined by phenol sulphuric acid method [43] and Kjeldahl method [44], respectively. The monosaccharide composition of the EPS fractions was measured using HPLC as reported by Siu et al. [45]. The EPS samples were hydrolyzed using 2 M trifluoroacetic acid, and the hydrolysates were reacted with 0.5 M 1-phenyl-3-methyl-5-pyrazolone (PMP) to produce PMP-labeled monosaccharides [46]. Determination of the PMP-labeled monosaccharides was carried out using an Agilent ZORBAX ECLIPSE XDB—C18 column on an Agilent 1100 system (Agilent Technologies, Santa Clara, CA, USA) coupled with a UV detector. The UV absorption was monitored at 250 nm. The molecular weight (MW), root-mean-square radius (Rm), molecular conformation and second virial coefficient (A_2_) were measured by gel permeation chromatograph conjugated with multi angle laser light scattering and ultraviolet detector (GPC-MALLS-UV) according to the method reported previously [47]. The GPC-MALLS-UV system was composed of a Waters e2695 HPLC system (Waters China Ltd., Hong Kong) equipped with a TSK-GEL G4000PWxl column and a TSK-GEL 5000PWxl column from Waters, a Waters 2414 refractive index detector, a Waters 2998 photodiode array detector, and a DAWN HELEOS II multi-angle (18 angles) laser light scattering detector (Wyatt Technology, Santa Barbara, CA, USA). An aliquot of 100 µL 0.2 mg/mL EPS sample solution was injected into the column and then eluted with 50 mM sodium nitrate solution containing 0.02 *w*/*v* sodium azide at a flow rate of 0.4 mL/min at 25 °C. The data from detectors were collected and analyzed using Astra 6.0 software (Wyatt Technology).

### 3.4. Preparation of AgNPs with EPS Fractions

The AgNPs were synthesized by reduction of AgNO_3_ using the prepared EPS fractions according to the procedure reported by Chen et al. [18] with minor modification. Two milliliters of 10 mM AgNO_3_ solution was mixed with an equal volume of 0.4 mg/mL EPS solution. The pH of the mixture was adjusted to pH 3.0, 4.0, 5.5, 7.0, 8.0, 9.0, and 10.0 respectively using 1 M HNO_3_ and 1 M NaOH solutions. The reaction mixture was heated in a glycerol bath at 100 °C for 240 min with constant stirring. The AgNP dispersion was then cooled in a 21 °C water bath and stored at room temperature before use. During the preparation and storage, the containers were covered with aluminum foil to prevent light exposure. 

### 3.5. UV-Vis Spectrum Measurements

A 8453 UV/Vis spectrometer (Agilent Technologies Hong Kong Ltd., Hong Kong) was used to record the UV-Vis spectra of reaction mixture samples from 300 nm to 800 nm. A two milliliter sample was taken from the reaction mixture every 30 min for UV-Vis measurement. The reaction mixture solution prior to thermal treatment was used as blank.

### 3.6. Transmission Electron Microscope (TEM)

Transmission Electron Microscopy was used to observe the morphology and analyze the elemental composition of the AgNPs [13]. A drop of AgNP dispersion was placed onto a carbon-coated 300 mesh copper grid and the grid was air-dried. The prepared samples were observed on a transmission electron microscope (JEM-2011 Electron Microscope, JEOL USA, Inc., Peabody, MA, USA) at an accelerating voltage of 200 kV. Images were recorded with a Gatan MultiScan 794 Camera and processed with Gatan Digital Micrograph 3.1 software package (Gatan, Pleasanton, CA, USA) and the particle size of AgNPs was measured at the same time. In addition, an energy dispersive spectrometer attached to the TEM system was used to obtain the energy dispersive spectroscopy (EDS) of AgNP during TEM observation for element content analysis of AgNP dispersions.

### 3.7. Dynamic Light Scattering Measurement

The EPS fractions and AgNP dispersions were measured using dynamic light scattering for their particle size [48]. The EPS fractions were dissolved in distilled water at 0.2 mg/mL before measurement. Particle size analysis was performed using a DynaPro NanoStar instrument (Wyatt Technology) with 632.8 nm wavelength and 90° scattering angle at 25 °C. Five replicates were conducted for each sample.

### 3.8. Statistical Analysis

All experiments were carried out in triplicates unless specified. The results were expressed as the mean and the standard deviation. Analysis of variance (ANOVA) was performed through the SPSS 16.0 software (SPSS Inc., Chicago, IL, USA).

## 4. Conclusions

In this study, AgNPs were synthesized using different EPS fractions from the fermentation broth of Cs-HK1 fungus under several pH conditions. The pH and molecular properties of EPS were important factors affecting the rate and yield of AgNPs formation and the properties of the AgNPs. Because of interactions between AgNPs and EPS molecules, the AgNPs were capped to form EPS-AgNP complexes. The results have revealed the relationship between the properties of AgNPs formed and the properties of the polysaccharide used for the formation of AgNPs. This relationship is useful for optimizing the conditions and polysaccharide materials for the synthesis of AgNPs with desired properties. In future studies, it is important to investigate the specific interactions between EPS and AgNP and the possible adsorption of Ag ions to and their release from the EPS molecules throughout the AgNP synthesis process, and the specific functional groups responsible for these interactions using X-ray photoelectron spectroscopy and X-ray diffraction in combination with FTIR and TEM analysis.

## Figures and Tables

**Table 1:** Composition and properties of EPS fractions.

EPS Fractions	Composition (Man:Glc:Gal)	MW (kDa) ^1^	PD ^2^	Rm (nm) ^3^	Conformation (Plot Slope)	A_2_ (1 × 10^−3^ mol·mL/g^2^)
P_0.5_	1.3:10.8:1	1022 ± 41.9	5.170 ± 0.38	147.3 ± 5.60	Random coil (0.50 ± 0.01)	−0.51 ± 0.02
P_2.0_	16:1:7.3	85.16 ± 4.34	10.8 ± 0.43	31.0 ± 1.30	Compact sphere (0.25 ± 0.02)	4.85 ± 0.04
P_5.0_	15:1:15.5	21.67 ± 0.85	3.287 ± 0.12	18.2 ± 1.80	Compact sphere (0.21 ± 0.03)	8.79 ± 0.05

^1^ MW: molecular weight; ^2^ PD: polydispersity; ^3^ Rm: root-mean-square radius.

**Table 2:** Elemental composition of EPS-AgNPs (by energy dispersive spectroscopy).

EPS-AgNPs	pH	Atom Contents (weight%)			
		C	N	O	Ag
P_0.5_-AgNPs	5.5	34.6 ± 3.32	6.76 ± 2.13	2.23 ± 0.96	56.4 ± 3.89
	7.0	33.9 ± 2.98	6.55 ± 2.43	2.05 ± 1.03	57.5 ± 4.41
	8.0	32.8 ± 3.07	7.03 ± 2.18	1.94 ± 1.27	58.2 ± 3.76
P_2.0_-AgNPs	5.5	14.9 ± 1.98	10.0 ± 1.87	2.67 ± 0.89	72.4 ± 3.65
	7.0	13.1 ± 2.04	11.4 ± 1.74	2.98 ± 0.93	73.5 ± 2.97
	8.0	11.5 ± 1.97	11.7 ± 1.89	2.74 ± 1.01	74.1 ± 3.06
	4.0	9.47 ± 1.04	10.2 ± 1.76	1.87 ± 1.05	78.5 ± 4.43
P_5.0_-AgNPs	5.5	7.34 ± 1.16	10.4 ± 2.03	1.89 ± 1.21	80.3 ± 3.57
	7.0	6.28 ± 1.21	10.1 ± 2.17	2.15 ± 0.99	81.5 ± 3.29
	8.0	7.11 ± 1.18	10.1 ± 1.87	2.03 ± 1.06	80.8 ± 3.87

## Supplementary Materials

Supplementary materials can be accessed at: http://www.mdpi.com/1420-3049/22/1/50/s1.
