# Supplementary material for: Formation and Physiochemical Properties of Silver Nanoparticles with Various Exopolysaccharides of a Medicinal Fungus in Aqueous Solution
Source: Molecules. 2016 Dec 29;22(1):50. doi: 10.3390/molecules22010050 (PMC6155925; doi:10.3390/molecules22010050)
Supplement: Supplementary file 1 [file molecules-22-00050-s001.pdf]

Supplementary Materials: Formation and Physiochemical Properties of Silver Nanoparticles with Various Exopolysaccharides of a Medicinal Fungus in Aqueous Solution

Wenjie Jian, Lu Zhang, Ka-Chai Siu, Angxin Song and Jian-Yong Wu

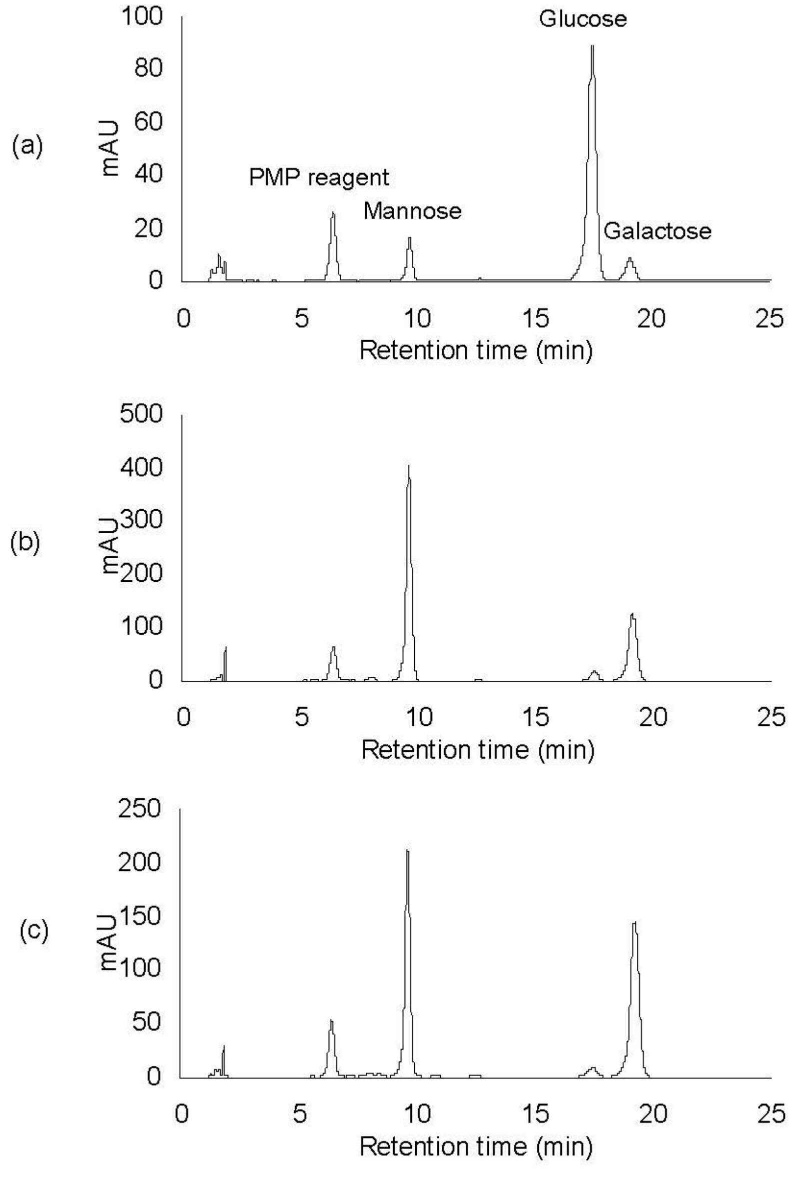

Figure S1. HPLC analysis on monosaccharide of $P_{0.5}$ (**a**); $P_{2.0}$ (**b**); and $P_{5.0}$ (**c**).

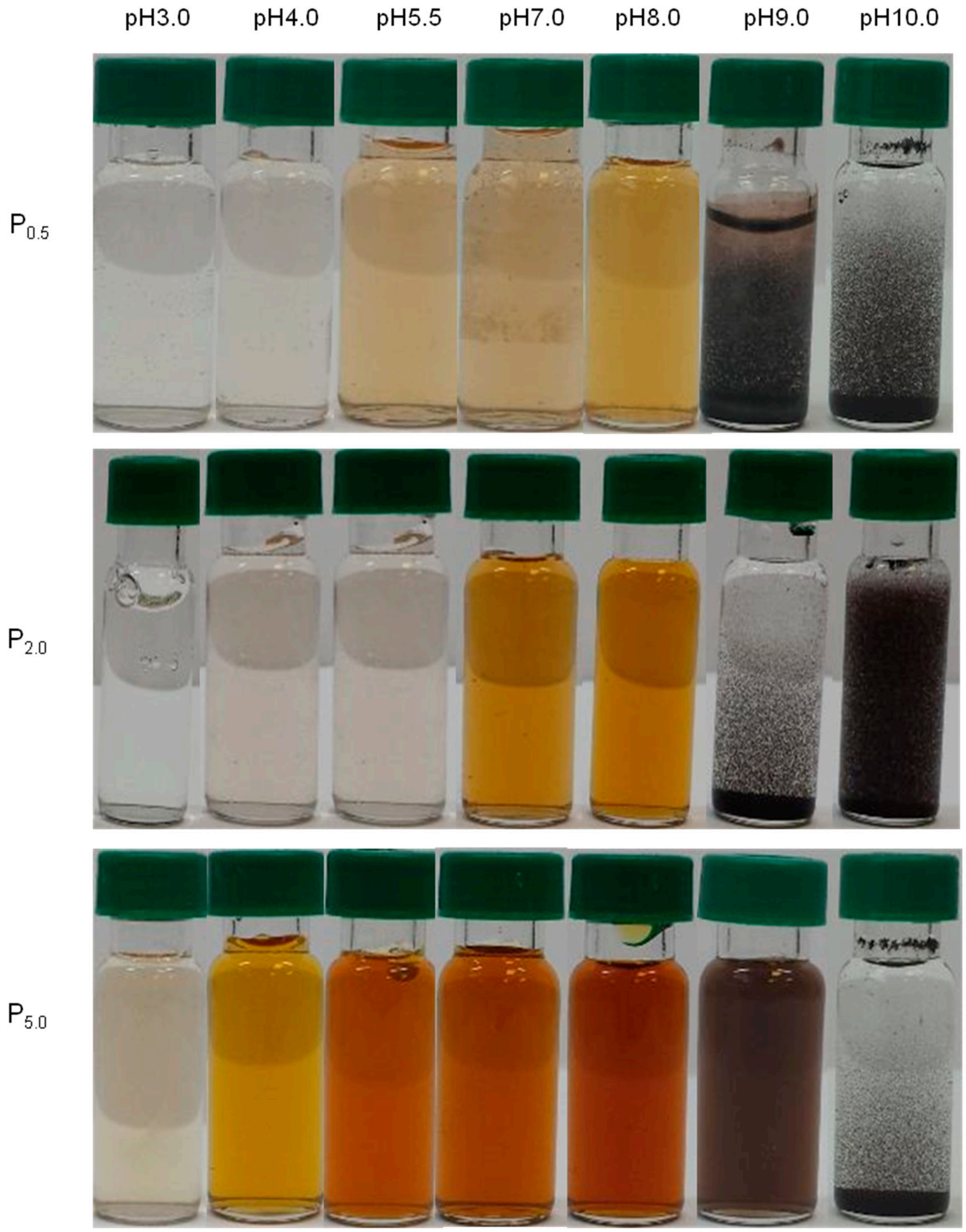

Figure S2. Photographs of $AgNO_3$ and EPS ($P_{0.5}$, $P_{2.0}$, and $P_{5.0}$) mixture solution at pH 3.0, 4.0, 5.5, 7.0, 8.0, 9.0, and 10.0 after heating at 100 °C for 240 min.

(a)

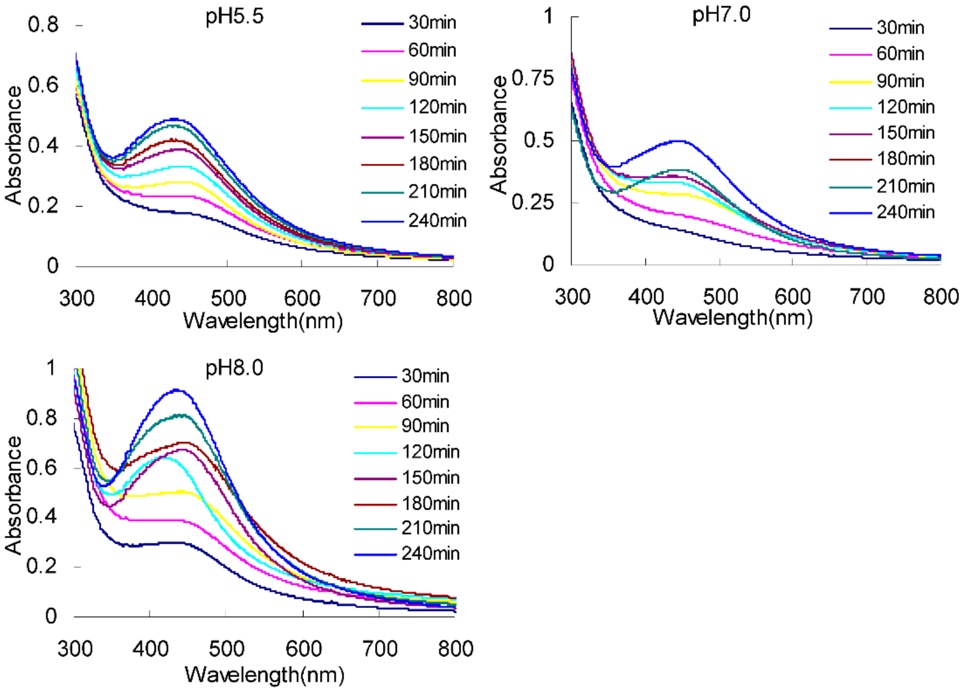
pH5.5
pH7.0
pH8.0
Absorbance
Wavelength(nm)
30min
60min
90min
120min
150min
180min
210min
240min

(b)

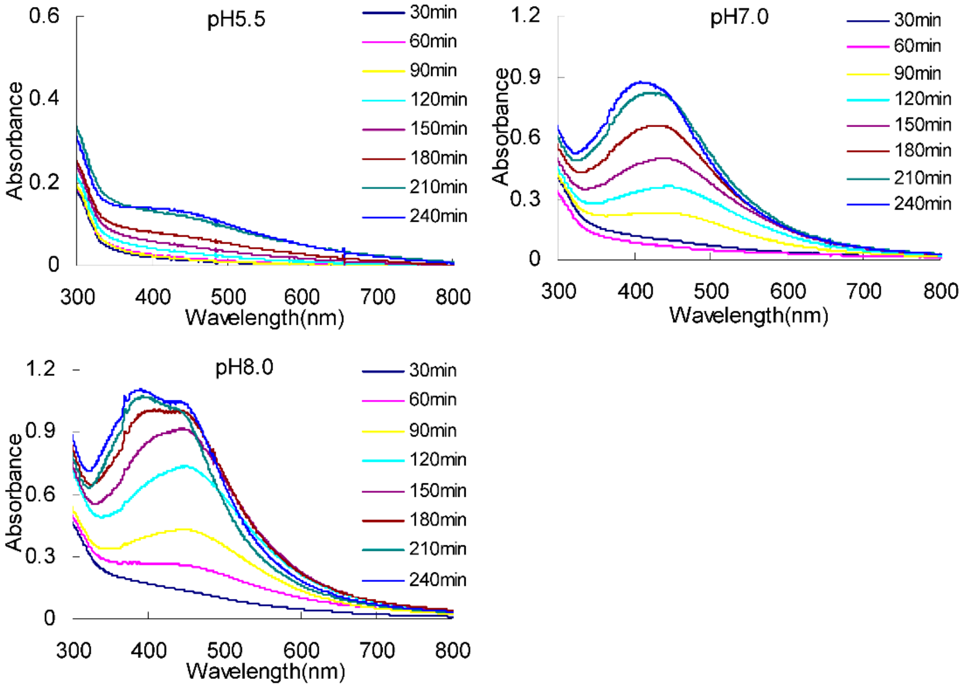
pH5.5
pH7.0
pH8.0
Absorbance
Wavelength(nm)
30min
60min
90min
120min
150min
180min
210min
240min

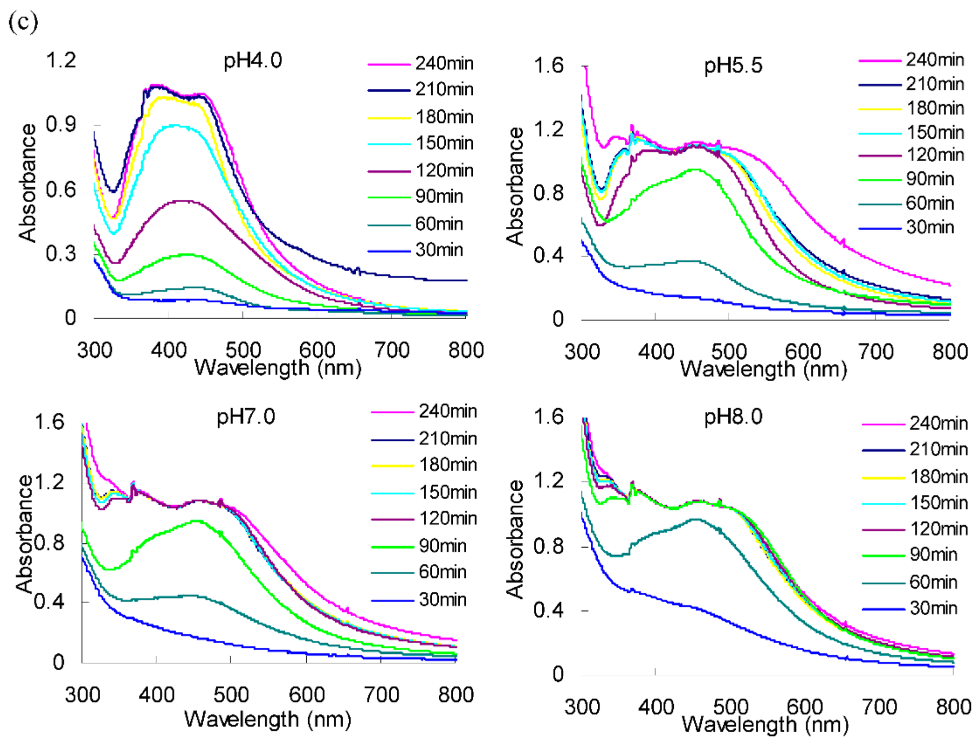

Figure S3. UV-vis spectra of the mixture of $AgNO_3$ and $P_{0.5}$ (**a**); $P_{2.0}$ (**b**); and $P_{5.0}$ (**c**) during the reaction process.

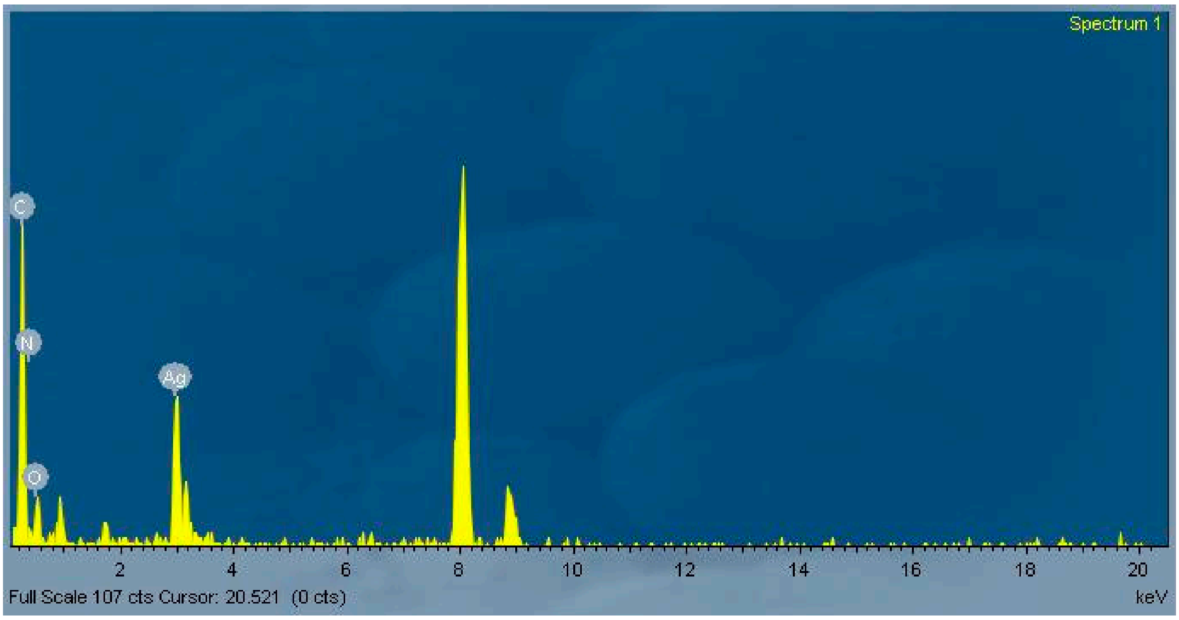

Figure S4. The energy dispersive spectroscopy spectrum of $P_{2.0}$-AgNP prepared at pH 8.0.

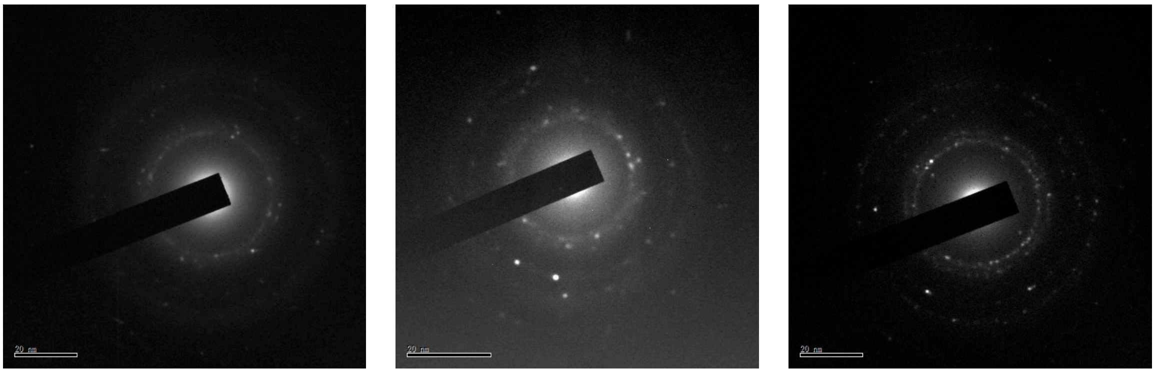

(**a**) (**b**) (**c**)

Figure S5. Selected area electron diffraction patterns (SAED) in TEM of (**a**) $P_{0.5}$-AgNPs; (**b**) $P_{2.0}$-AgNPs; and (**c**) $P_{5.0}$-AgNPs prepared at pH 8.0.
